# A comparative study of mortality differences and associated characteristics among elderly and young adult patients hospitalised with COVID-19 in India

**DOI:** 10.1186/s12877-023-03955-6

**Published:** 2023-04-25

**Authors:** Kartik Mittal, Minakshi Dhar, Monika Pathania, Dipesh Jha, Vartika Saxena

**Affiliations:** 1 Department of Geriatric Medicine, All India Institute of Medical Sciences, Rishikesh, India; 2 Department of Community and Family Medicine, All India Institute of Medical Sciences, Rishikesh, India

**Keywords:** COVID, Geriatric, Elderly, COVID mortality, COVID deaths, COVID in India

## Abstract

**Introduction:**

Studies have shown that elderly have been disproportionately impacted by COVID pandemic. They have more comorbidities, lower pulmonary reserve, greater risk of complications, more significant resource utilization, and bias towards receiving lower-quality treatment.

**Objectives:**

This research aims to determine the characteristics of those who died inhospital due to COVID illness, and to compare these factors between elderly and young adults.

**Methods:**

We conducted a large retrospective study at a government run center in Rishikesh, India, from 1^st^ May 2020 till 31^st^ May 2021, and divided study population into adults (aged 18 to 60 years) and elderly (aged 60 years). We evaluated and compared our data for presenting symptoms, vitals, risk factors, comorbidities, length of stay, level of care required, and inhospital complications. Long-term mortality was determined using telephonic follow-up six months after discharge.

**Results:**

Analysis showed that elderly had 2.51 more odds of dying inhospital compared to younger adults with COVID. Presenting symptoms were different for elderly COVID patients. The utilization of ventilatory support was higher for elderly patients. Inhospital complications revealed similar profile of complications, however, kidney injury was much higher in elderly who died, while younger adults had more Acute Respiratory Distress. Regression analysis showed that model containing cough and low oxygen saturation on admission, hypertension, Hospital Acquired Pneumonia, Acute Respiratory Distress Syndrome, and shock, predicted inhospital mortality.

**Conclusion:**

Our Study determined characteristics of inhospital and long-term mortality in elderly COVID patients and compared them from adults, to help better triaging and policy making in future.

## Take home message

This study, and its detailed analysis, has shown how various characteristics like risk factors, comorbidities, level of care received, and inhospital complications have disproportionately affected elderly patients with COVID in terms of both short term inhospital mortality, as well as long term post discharge survival. We hope that this data will be beneficial for policy makers all over the world to be better prepared for resource allocations in upcoming pandemic like the present one, and for clinicians to be able to better identify profile of elderly COVID patients who are more likely to get sicker, and have a risk of higher mortality, so that early, and a more aggressive care can be given to these selected group of elderly COVID patients based on their presenting profile, and thus improve outcomes. We also hope this data can be used by researchers all over world to compare COVID characteristics of Indian population with their respective study groups.

## Background

The COVID-19 pandemic [1–3] has disproportionately affected elderly patients worldwide, with higher rates of complications [4–12], mortality, and resource utilization. Elderly patients with pre-existing comorbidities and poor pulmonary function are at particular risk, and may receive lesser quality of care due to resource constraints in overburdened healthcare systems [13–31]. In resource-limited countries like India, where healthcare systems were not nationalized or funded during the pandemic, the vulnerability of elderly patients is of particular concern [30]. This study aims to establish the burden of mortality and resource utilization among vulnerable elderly patients in India who were hospitalized due to COVID-19. We will characterize and compare risk factors, comorbidities, and complications among those who died in hospital and those who were successfully discharged, with a focus on identifying elderly patients who might benefit from early triaging and admission to intensive care units. This data will help healthcare systems better allocate resources in pandemic-hit regions. We will also follow up with discharged patients for up to six months to assess their long-term survival. This single-center study from a central government institute in India will contribute to a better understanding of the impact of COVID-19 on vulnerable elderly patients in developing nations.

### Research gap

The COVID-19 pandemic has significantly impacted the elderly population [13–31], and studies have shown that they are at a higher risk of severe illness and death due to the virus [14–30]. However, there is a lack of research focusing specifically on the characteristics of those who died inhospital due to COVID illness and the comparison of these factors between elderly and young adults. This study aims to address this research gap by identifying the specific factors that contribute to inhospital mortality in elderly COVID patients and comparing them to those of younger adults. Previous studies have highlighted the vulnerability of the elderly population to COVID-19, but the specific characteristics of elderly patients who die inhospital have not been well defined. In addition, there is limited research that compares the characteristics of elderly and young adult patients who die from COVID-19, which can inform targeted interventions for these two distinct age groups. Therefore, the current study contributes to the literature by addressing these research gaps and providing important insights into the factors that contribute to inhospital mortality in elderly COVID patients. The identification of these factors is critical for improving the management of elderly COVID patients and guiding policy-making decisions to better allocate healthcare resources. By comparing the characteristics of elderly and young adult COVID patients who died inhospital, this study helps to identify age-specific factors that may be targeted in future interventions. Therefore, the research question for this study is: What are the specific factors that contribute to inhospital mortality in elderly COVID patients, and how do these factors compare to those of younger adults with COVID-19? Overall, this study contributes to the literature by providing insights into the characteristics of inhospital mortality in elderly COVID patients and comparing them to those of younger adults. The results of this study can inform targeted interventions and policies to improve the management of COVID-19 patients and reduce mortality rates, particularly in vulnerable elderly populations.

## Methods

**Fig. 1:**
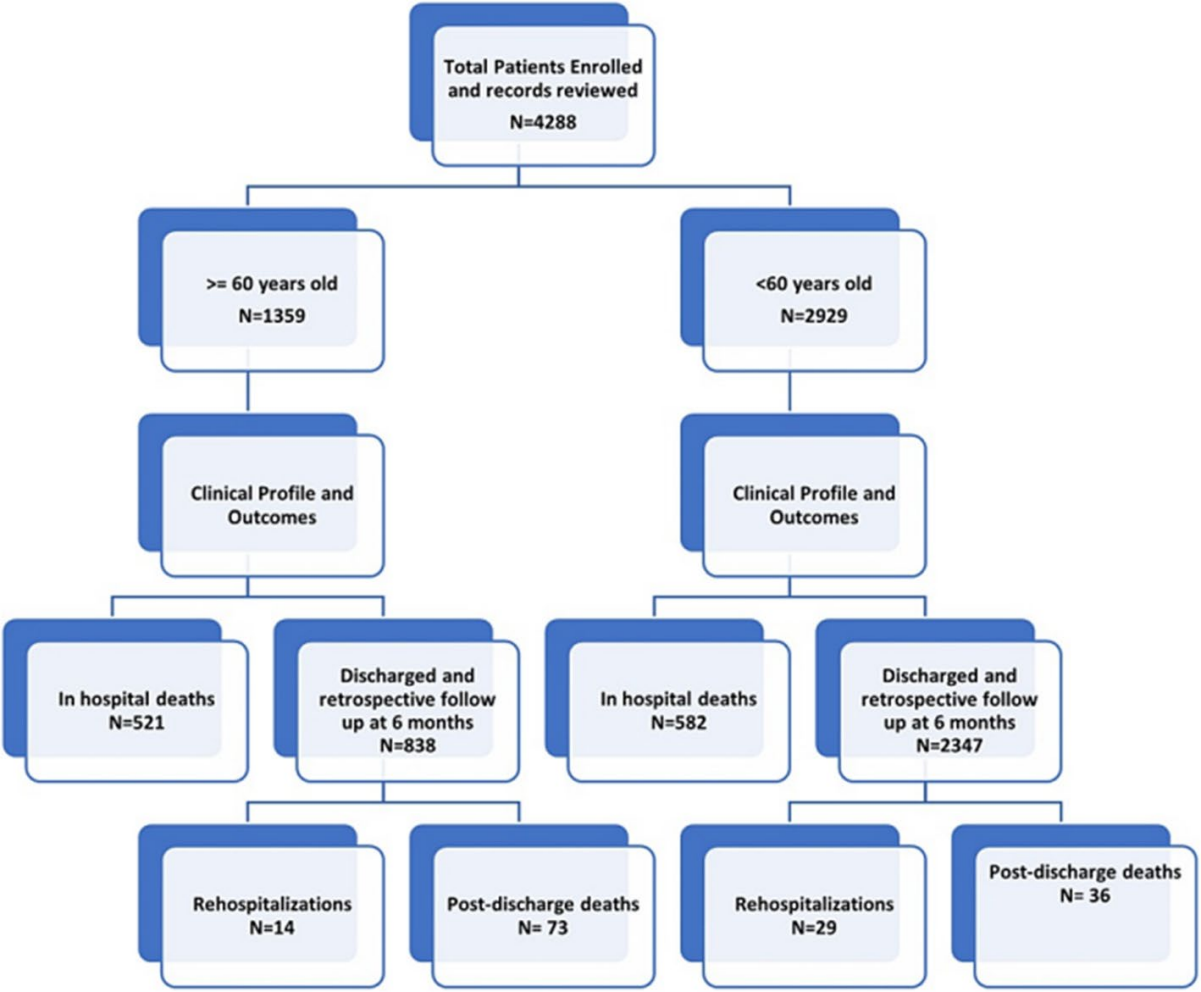
Study flowchart

This is a large, single center, retrospective analysis from a tertiary care hospital in North Indian city of Rishikesh. This center functions under central government, and was fully converted to a tertiary referral facility exclusively for COVID patients during both first and second waves of pandemic in India. This study took data of all adult hospitalised patients at this center from,1^st^ May 2020 to 31^st^ May 2021, and retrospectively studied the clinical profile and outcomes, including mortality, demographics, risk factors, comorbidities, duration of stay, level of care needed, and inhospital complications, and for the purpose of our study, we divided our participants into two age groups: adults (age 18 to < 60 years) and elderly( age ≥ 60 years) as per WHO definition of elderly in developing countries. A retrospective telephonic followup after 6 months of discharge was also done to determine long term mortality.(refer to Fig. 1 study flowchart) The data was cleaned and coded by trained personnel to ensure consistency and accuracy. Missing data was reported and excluded from the analysis. Logistic regression analysis was used to determine the association between risk factors and mortality. Multi-collinearity was checked using variance inflation factor (VIF) and no significant collinearity was found. COVID illness severity was operationalized according to standard definitions of mild, moderate, and severe. Statistical analyses were conducted using SPSS software.

## Results

The overall inhospital mortality in our single center study over a course of more than 12 months and spanning two waves of deaths was 38% in more than 60-year-old elderly patients, and 20% in less than 60-year-old adults. The odds ratio of more than 60 years olds for inhospital mortality compared those those younger was 2.51 (95% CI, 2.18–2.89). Table 1 shows comparison of demographic characteristics of COVID-19 patients who died in-hospital divided based on age groups, with their mean age, gender distribution and risk factors. The mean age of hospitalised elderly who died inhospital was 69.6 years compared to 45 years in young adult group. In both groups, majority of those who died were males. Smokers who were aged had a higher mortality than those younger (17% vs 13%), but conversely, alcoholics who were younger had a high chance of death (5% vs 8%).

**Table 1:** Comparison of In-Hospital Mortality in both age groups for demographic characteristics and duration of stay

Parameters	In-Hospital Mortality		*p* value
	** < 60 years** **(*n* = 582)**	** ≥ 60 years** **(*n* = 521)**	
**Age (Years)**	45.08 ± 10.55	69.66 ± 7.67	< 0.001
**Gender**			0.429
Male	376 (64.6%)	359 (68.9%)	
Female	206 (35.4%)	162 (31.1%)	
**Smoker**	78 (13.4%)	89 (17.1%)	< 0.001
**Alcoholic**	46 (7.9%)	27 (5.2%)	< 0.001
**Duration Of Stay (Days)**	8.96 ± 6.46	9.59 ± 7.12	< 0.001

This Table also shows the mean duration of stay in days for those who died in hospital from COVID-19, analyzed retrospectively and distributed based on age groups, and reveals a need for longer duration of stay for those more than 60 years old. In those who had died, mean days of stay for more than 60 years was 9.5 ± 7.1 days, as compared to adult group having 8.9 ± 6.4 days. Even after utilising more days on hospital bed, elderly COVID patients have more overall mortality, emphasizing greater utilization of resources with poorer outcomes.

Table 2 shows distribution of comorbidities in those patients who died inhospital from COVID-19, and divided based on age groups. The most prevalent comorbidity in those above 60 years who died were hypertension (47%), diabetes (36%), heart disease (20%) and CKD (17%), while in those younger than 60 years, most prevalent in those who died were diabetes (34%) and hypertension (24%). Those who died with history of COPD were more in elderly group (9% vs 6%). Interestingly, death in most comorbid parameters was more in elderly group, except CLD (2% vs 6%), where death in those younger than 60 years was more.

**Table 2:** Comparison of In-Hospital Mortality in both age groups for comorbidities

**Parameters**	**In-Hospital Mortality**		
	** < 60 years** **(*n* = 582)**	**≥ 60 years** **(*n* = 521)**	*p* value
**Diabetes**	200 (34.4%)	185 (35.5%)	< 0.001
**Hypertension**	142 (24.4%)	244 (46.8%)	< 0.001
**Heart Disease**	43 (7.4%)	106 (20.3%)	< 0.001
**COPD**	33 (5.7%)	46 (8.8%)	0.002
**CKD**	40 (6.9%)	89 (17.1%)	< 0.001
**CLD**	34 (5.8%)	8 (1.5%)	< 0.001
**Cancer**	18 (3.1%)	14 (2.7%)	0.031
**Stroke**	19 (3.3%)	21 (4.0%)	< 0.001
**BASED ON NUMBER OF COMRBIDITIES**			
**No comorbidities**	300 (51.5%)	169 (32.4%)	< 0.001
**Only one comorbidity**	147 (25.2%)	146 (28%)	< 0.001
**Two comorbidities**	92 (15.8%)	131 (25.1%)	< 0.001
**More than two comorbidities**	43 (7.4%)	75 (14.3%)	< 0.001

The death rate of those having no comorbidities was more in less than 60-year group (32% elderly vs 52% younger), showing that even in non-morbid young individuals, COVID had the serious ability to cause mortality. For all “more the one” number of comorbidities groups, mortality was understandably more in older adults with multiple comorbidities. The death in elderly with one comorbidity (28% vs 25%), two comorbidities (25% vs 16%), and more than two comorbidities (14% vs 7%) was much more significant in elderly than their younger comorbid counterparts, with the gap in mortality widening as the number of comorbidities increased.

Table 3 shows utilization of type of ventilatory support and level of care- divided into “ward based” and “critical care unit” requirement. At our center, all those who maintained well on oxygen alone were managed in wards, and those who needed either NIV(non-invasive ventilation) or MV (mechanical ventilation) were shifted to critical care unit. This table reveals these requirements retrospectively in those who died of COVID-19 divided based on age, and shows higher need for “critical care based” need for those more than 60 year old, suggesting a need for higher level of care inhospital for this elderly age group.

**Table 3:** Comparison of In-Hospital Mortality in both age groups for type of ventilatory support needed

**Parameters**	**In-Hospital Mortality**		
	** < 60 years** **(*n* = 582)**	** ≥ 60 Years** **(*n* = 521)**	*p* value
**Type of Ventilatory Support**			< 0.001
None/Ward-Based	32 (5.5%)	18 (3.5%)	
1) None	27 (4.6%)	12 (2.3%)	
2) O2	5 (0.9%)	6 (1.2%)	
NIV/MV	550 (94.5%)	503 (96.5%)	
1) NIV	62(10.7%)	54 (10.4%)	
2) MV	488 (83.8%)	449 (86.2%)	
**NIV Outcome**			0.269
Success	62 (16.4%)	54 (14.9%)	
Failed	315 (83.6%)	309 (85.1%)	

Similarly, NIV success was defined as those who did not further needed MV support, and managed with NIV alone, and success for this was higher for those less than 60 years, however, the overall success rate was poor for both age groups.

While the utilization of ventilatory support was much higher for older patients in the overall data of hospitalised patients, in those who died, the utilization was comparable for both NIV (10% vs 11%) and MV (86% vs 84%) groups. This might be used as an argument to highlight, that even though utilization of resources was higher in elderly patients, those who died had equal chance of being of any age group when offered highest level of ventilatory support.

Table 4 shows the list of most prevalent inhospital complications that were retrospectively analyzed in those who died inhospital of COVID-19, distributed based on age. It reveals that the most prevalent inhospital complication was ARDS in those who died, and was comparable in both age groups. However, prevalence of HAP/VAP, AKI and shock was more in more than 60-year-old age group in those who died inhospital.

**Table 4:** Comparison of In-Hospital Mortality in both age groups for in-hospital complications

**Parameters**	**In-Hospital Mortality**		
	** < 60 years** **(*n* = 582)**	** ≥ 60 years** **(*n* = 521)**	*p* value
**HAP/VAP**	218 (37.5%)	229 (44.0%)	< 0.001
**ARDS**	424 (72.9%)	385 (73.9%)	< 0.001
**AKI**	93 (16.0%)	156 (29.9%)	< 0.001
**Shock**	135 (23.2%)	152 (29.2%)	< 0.001
**PTE**	38 (6.5%)	16 (3.1%)	0.014
**Mucormycosis**	22 (3.8%)	10 (1.9%)	0.32

Inhospital complications analysis revealed the most common complications factors leading to death in order of prevalence for both groups were ARDS (74% VS 73%), HAP/VAP (44% VS 38%), Shock (29% VS 23%) and AKI (30% VS 16%), although the prevalence of HAP/VAP, AKI and shock was much higher in those more than 60 years who died, as compared to younger adults, in whom ARDS was disproportionately more than any other complication in causing mortality. The prevalence of PTE was higher in adults than elderlies who died (3% vs 7%).

In both age groups, cough (99% vs 96%) and shortness of breath (98% vs 95%) at admission at higher chance of dying, simply owing to more chances of COVID Pneumonia and oxygen requirement as expected with this symptomatology. However, like the overall hospitalization data, even among those who died, older patients had less chance of presenting with fever (79% vs 99%), thus making fever a poor marker of prognosis in both age groups.

Even though pattern of distribution of vitals was similar in those who died, showing higher temperature (103.48 ± 1.03 vs 103.41 ± 0.98), systolic blood pressures (102 ± 31 vs 100 ± 27), diastolic blood pressure (65 ± 17 vs 64 ± 16), heart rate (137 ± 17 vs 136 ± 14) and lower spo2 (83.7 ± 3.1 vs 83.9 ± 3.3) in older patients, the difference lies in comparisons from the vitals of overall data. To recapitulate, the overall data showed mean temperature (101.9 ± 2.9 vs 101.5 ± 1.3), mean heart rate (123 ± 16 vs 120 ± 12), systolic blood pressure (123 ± 28 vs 117 ± 18), diastolic blood pressure (77 ± 16 vs 76 ± 12), and lower admission SpO2 (88 ± 4 vs 89 ± 3). Thus, those who died had higher mean temperature values, higher mean heart rates, lower systolic and diastolic blood pressures, and lower SpO2 measure on admission.

Table 5 summarizes the regression analysis for a model created to include the most statistically significant data from descriptive data. A univariate analysis of these factors is done to calculate respective univariate Odds Ratio. A multivariate analysis of the most significant univariate factors is done to determine the multivariate odds ratio for in-hospital mortality. Thus, the most important factors in this analysis for mortality were pre-existing hypertension, cough with low spo2 on admission, Severe Covid pneumonia, and inhospital development of HAP/VAP, ARDS or shock.

**Table 5:** Risk Factors for in-hospital mortality using logistic regression analysis

In-Hospital Mortality	OR (univariable)	OR (multivariable)
≥ 60 Years	2.54 (2.19–2.93, *p*** < 0.001**)	1.38 (0.91–2.11, *p* = 0.132)
Smoker	1.72 (1.40–2.11, *p* < **0.001**)	1.05 (0.48–2.27, *p* = 0.898)
Alcoholic	1.85 (1.36–2.50, *p* < **0.001**)	0.99 (0.26–3.48, *p* = 0.988)
Diabetes	2.26 (1.94–2.64, *p* < **0.001**)	1.18 (0.76–1.84, *p* = 0.453)
Hypertension	1.53 (1.32–1.78, *p* < **0.001**)	0.44 (0.27–0.70, *p* = **0.001**)
Heart Disease	2.65 (2.11–3.34, *p* < **0.001**)	1.44 (0.73–2.78, *p* = 0.287)
COPD	1.48 (1.11–1.96, *p* = **0.007**)	1.30 (0.50–3.33, *p* = 0.589)
CKD	2.45 (1.92–3.12, *p* < **0.001**)	1.23 (0.50–3.03, *p* = 0.648)
CLD	1.89 (1.26–2.82, *p* = **0.002**)	1.60 (0.34–7.94, *p* = 0.556)
HAP/VAP	3.60 (3.09–4.20, *p* < **0.001**)	2.96 (1.90–4.64, *p*** < 0.001**)
ARDS	14.15 (11.96–16.78, *p*** < 0.001**)	2.25 (1.34–3.84, *p* = **0.003**)
AKI	2.88 (2.39–3.48, *p* < **0.001**)	1.80 (0.86–3.75, *p* = 0.120)
Shock	17.00 (12.92–22.69, *p* < **0.001**)	10.38 (4.59–24.00, *p*** < 0.001**)
Cough	29.28 (19.72–45.82, *p* < **0.001**)	0.02 (0.00–0.21, *p* = 0.**005**)
SOB	77.45 (56.46–109.45, *p* < **0.001**)	1.75 (0.60–5.14, *p* = 0.306)
Systolic BP (mmHg) (Admission) Mean (SD)	0.93 (0.93–0.94, *p* < **0.001**)	0.99 (0.99–1.00, *p* = 0.122)
SpO2(ORA) (Admission) Mean (SD)	0.33 (0.31–0.36, *p* < **0.001**)	0.39 (0.35–0.43, *p* < **0.001**)

Our logistic regression analysis showed that in a univariate analysis, in a model composed of Age (> 60 years), Risk Factor (smoker/alcoholic), diabetes, hypertension, heart disease, COPD, CKD, CLD, COVID severity (moderate/severe), inhospital complications (HAP, ARDS, AKI, shock), cough or SOB at admission and systolic BP and SpO2 on admission had the most statistically significant potential to predict inhospital mortality in hospitalised patients with COVID-19.

Table 6 shows the demographic characteristics and risk factors of having post-discharge mortality and distributed based on age group (this data was retrospectively collected telephonically 6 months after discharge). Total 73 patients in elderly group and 36 patients in adult group had died on follow-up. It reveals that mean age of those died after discharge in more than 60 years old group was around 67 years, comparable to those who died in hospital. There is a higher rate of death in males in both age groups. The odds ratio for post-discharge mortality in more than 60 years olds compared to younger adults was 9.43 (95% CI, 6.25–14.23).

**Table 6:** Comparison between Post-Discharge Mortality and baseline demographic characteristics in both age groups

**Parameters**	**Post-Discharge Mortality**		
	** < 60 years** **(*n* = 36)**	** ≥ 60 Years** **(*n* = 73)**	
**Age (Years)**	46.03 ± 10.84	67.23 ± 6.13	0.004
**Gender**			0.535
Male	22 (61.1%)	43 (58.9%)	
Female	14 (38.9%)	30 (41.1%)	
**Smoker**	4 (11.1%)	5 (6.8%)	0.609
**Alcoholic**	5 (13.9%)	1 (1.4%)	0.334

Table 7 shows the 6-month post discharge mortality and its retrospective analysis to determine their respective inhospital complications when they were admitted, and reveals in more than 60 year olds who died, inhospital AKI f/b HAP/VAP recovered patients had the highest prevalence of death on follow up after discharge, while most of the less than 60 years old who died on follow up had ARDS or HAP/VAP during inhospital stay. For more than 60 years olds, it was AKI (37%) that had worst long-term outcomes, much more than HAP/VAP (34%) or ARDS (25%). The pattern of data was quite different in younger adults, where the most impactful parameter that determined post discharge mortality was ARDS (75%) during their index admission, followed by much distant and much less likely HAP/VAP (14%) in this age group.

**Table 7:** Association between Post-Discharge Mortality and inhospital complications, ventilatory support during admission and selected pre-existing comorbidities in both age groups

**Parameters**	**Post-Discharge Mortality**		
	** < 60 years** **(*n* = 36)**	** ≥ 60 years** **(*n* = 73)**	***p* value**
**Type of Ventilatory Support**			< 0.001
None/oxygen (Ward-Based)	3 (8.3%)	62 (84.9%)	
NIV/MV (critical care)	33 (91.7%)	11 (15.1%)	
**Inhospital complications**			
**HAP/VAP**	5 (13.9%)	25 (34.2%)	0.805
**ARDS**	27 (75.0%)	18 (24.7%)	< 0.001
**AKI**	2 (5.6%)	27 (37.0%)	0.377
**Pre-existing Comorbidities**			
**Hypertension**	12 (33.3%)	37 (50.7%)	0.011
**Heart Disease**	1 (2.8%)	12 (16.4%)	0.522
**COPD**	2 (5.6%)	1 (1.4%)	0.294
**CKD**	2 (5.6%)	12 (16.4%)	0.227

In those more than 60-year-olds most were hypertensive (51%) or diabetic (36%), while what was interesting was the prevalence of CKD (16%) comparable to heart disease (16%) patients in post-discharge risk of mortality.Among the less than 60-year-old adult group, the risk of both hypertensives (33%) and diabetics (31%) for dying after discharge was comparable. The post discharge mortality data was too low to make our data statistically significant in any of these groups, except for hypertension and diabetes.

These patients who had died after discharge, majority of those who were more than 60 years old had received ward-based care with o2 support (85%), while majority of those who were less than 60 years were managed in critical care unit (with NIV or MV) (92%). This observation might be explained by assuming that most elderly patients who had severe disease had died inhospital, and those who were younger than 60 years who had been discharged after severe disease with history of receiving some form of ventilatory support had damaged lungs which resulted in their post-discharge risk of respiratory morbidity and mortality.

Table 8 summarizes the regression analysis for a model created to include the most statistically significant data from descriptive data. A univariate analysis of these factors is done to calculate respective univariate Odds Ratio. A multivariate analysis of these significant univariate factors is done to determine the multivariate odds ratio for post-discharge mortality. The univariate analysis revealed that Age (> 60 years), diabetes, hypertension, heart disease, CKD, inhospital HAP/VAP, ARDS or AKI, and shortness of breath and systolic blood pressure on index admission had the highest risk of predicting the post-discharge mortality. The multivariate logistic regression was applied to these significant parameters, which revealed that after keeping these factors constant, a model comprising of Age (> 60 years), ARDS and AKI during inhospital stay had the highest potential to predict death after discharge on long term follow up in hospitalised COVID-19 patients.

**Table 8:** Risk Factors for Post-Discharge Mortality using logistic regression analysis

**Post-discharge mortality**	**OR (univariable)**	**OR (multivariable)**
≥ 60 Years	8.89 (5.85–13.75, *p*** < 0.001**)	4.60 (2.64–8.09, *p*** < 0.001**)
Diabetes	2.64 (1.72–3.99, *p* < **0.001**)	1.05 (0.63–1.72, *p* = 0.845)
Hypertension	3.12 (2.08–4.66, *p*** < 0.001**)	1.45 (0.88–2.39, *p* = 0.140)
Heart Disease	3.55 (1.84–6.35, *p* < **0.001**)	0.99 (0.46–2.03, *p* = 0.977)
CKD	3.48 (1.85–6.12, *p* < **0.001**)	0.82 (0.35–1.84, *p* = 0.635)
HAP/VAP	2.39 (1.52–3.68, *p*** < 0.001**)	1.34 (0.76–2.29, *p* = 0.297)
ARDS	5.16 (3.40–7.78, *p*** < 0.001**)	2.62 (1.52–4.51, *p* = **0.001**)
AKI	4.85 (3.03–7.57, *p*** < 0.001**)	2.02 (1.02–3.92, *p* = **0.040**)
SOB	16.58 (10.61–26.72, *p* < **0.001**)	7.93 (4.67–13.77, *p* < **0.001**)
Systolic BP (mmHg) (Admission)Mean (SD)	1.03 (1.02–1.04, *p* < **0.001**)	1.00 (0.99–1.02, *p* = 0.688)

## Discussion

The study examined the in-hospital mortality of COVID-19 patients in two age groups, more than 60 years and less than 60 years, and found that the mortality rate was higher in the elderly group. The study also revealed that elderly patients had a higher chance of comorbidities and lower chances of presenting with fever, despite having higher mean temperature values and higher mean heart rates on admission. The utilization of ventilatory support was higher in the elderly group, but the use of highest needed ventilatory support was comparable in both age groups. The most common complications leading to death in both groups were ARDS, HAP/VAP, Shock, and AKI, with the prevalence of HAP/VAP, AKI, and shock being higher in the elderly group, while ARDS was disproportionately higher in the younger adult group. We also discussed models to predict inhospital [32] and post-discharge [33, 34] mortality in COVID patients.

Even though our study had a large sample size, it did have certain limitations. Firstly, It was a single center study, so geographical diversity in our patient population was limited, which might not reflect the overall situation of COVID-19 patients in different regions and at different time points. Secondly, it’s a retrospective study of data, and future prospective studies with similar aims will have stronger base of evidence. Thirdly, patient follow-up [33, 34] was retrospective and one-time, due to resource constraints and poor compliance of patient re-visits in our setup, which could have been improved if a strong telemedicine follow-up system would have been in place. Additionally, the study did not examine the effect of vaccination status or the severity of the disease on the mortality rate, which could be important factors to consider.

Despite these limitations, the study successfully highlighted the higher mortality rate in elderly COVID-19 patients and the prevalence of comorbidities as an important factor contributing to mortality. The study also emphasized the importance of oxygen requirement and the need for higher ventilatory support in elderly patients. The findings of the study could be useful in improving the management of COVID-19 patients, especially the elderly population, by identifying the key factors that contribute to mortality.

In comparison to other studies, our study found a higher overall mortality rate in COVID-19 patients [14–31], especially in the elderly group. The study also revealed that the prevalence of comorbidities [35–42] and the utilization of ventilatory support [43–50] were higher in the elderly group than in other studies. However, the findings related to the most common complications leading to death were consistent with other studies [50–52]. Overall, the study contributes to the existing literature on COVID-19 and highlights the importance of age and comorbidities in determining the mortality rate in COVID-19 patients.

## Conclusion

The COVID-19 pandemic has had a disproportionate impact on elderly populations worldwide. In this retrospective study, we evaluated and compared the characteristics of COVID-19 patients who died inhospital between two age groups, adults aged 18–60 years and elderly aged 60 years and older. Our findings revealed that the odds of dying inhospital were 2.51 times higher in elderly patients than in younger adults. Additionally, elderly patients had different presenting symptoms and required a higher level of care, including a greater utilization of ventilatory support.

Our study fills a critical research gap in the current literature, which has primarily focused on the general characteristics and outcomes of COVID-19 patients. Our specific focus on the differences between elderly and younger adult COVID-19 patients provides a valuable contribution to the literature, as it highlights the unique challenges and risks that the elderly face during the pandemic. This information can inform public health policies and healthcare practices, particularly in resource-constrained settings, to better address the needs of this vulnerable population.

Our study has several important implications for the future. First, the findings of this study highlight the need for continued efforts to protect the elderly from COVID-19, particularly through targeted vaccination campaigns and other preventive measures. Second, our results underscore the importance of geriatric care and specialized training for healthcare providers who treat elderly COVID-19 patients. Finally, our study highlights the need for further research to better understand the underlying mechanisms of COVID-19 in elderly populations, particularly with regard to comorbidities and long-term outcomes.

In conclusion, our study provides valuable insights into the characteristics and outcomes of elderly COVID-19 patients who died inhospital, which can inform public health policies and healthcare practices in the ongoing effort to control the COVID-19 pandemic. As the pandemic continues to evolve, it is crucial to prioritize the needs of vulnerable populations, particularly the elderly, to ensure that healthcare resources are allocated effectively and efficiently.

The COVID-19 pandemic has been a global challenge that has required a collective effort to overcome. As we continue to navigate this crisis, we must prioritize the needs of the most vulnerable among us, particularly the elderly, to ensure that we emerge from this pandemic stronger and more resilient than ever before.

## Data Availability

Data was extracted using Medical Record Section of AIIMS Rishikesh, and using electronic medical record system called “e-Hospital” operated by Government of India. For any request regarding further details of data, kindly contact the corresponding author, Dr Kartik Mittal via email (drkartikmittal@yahoo.in).
